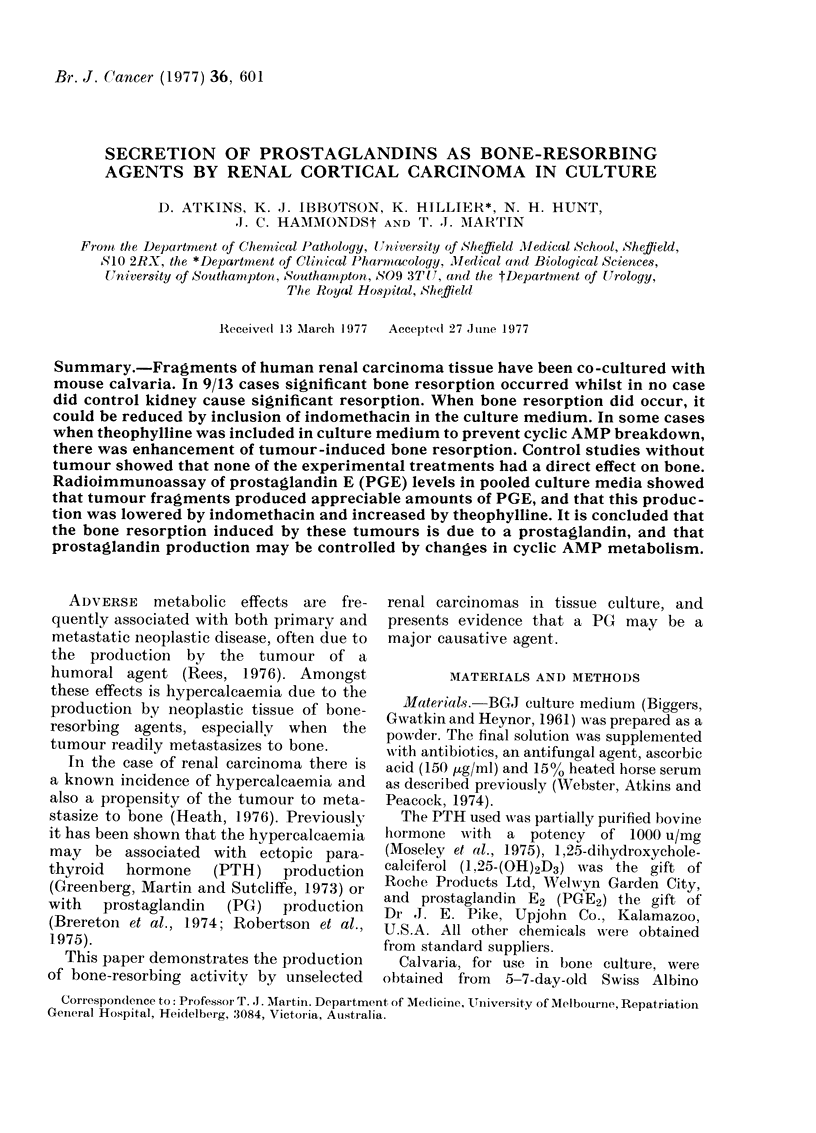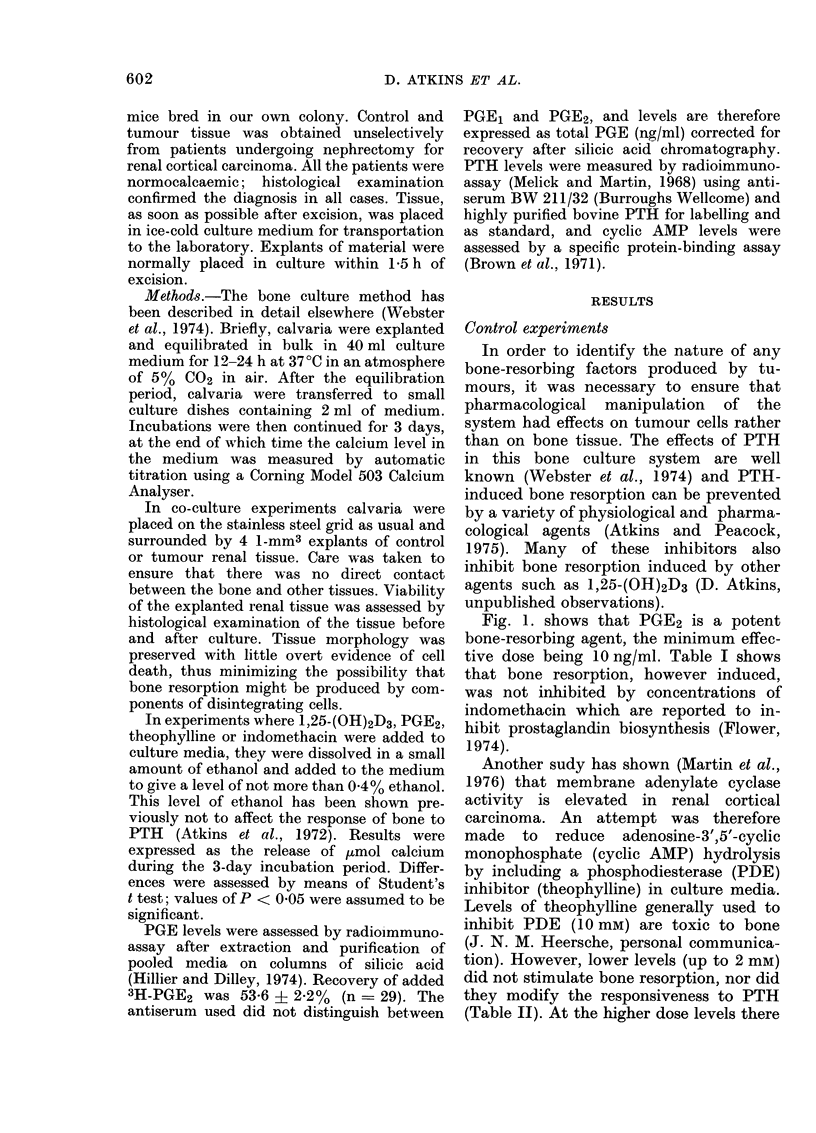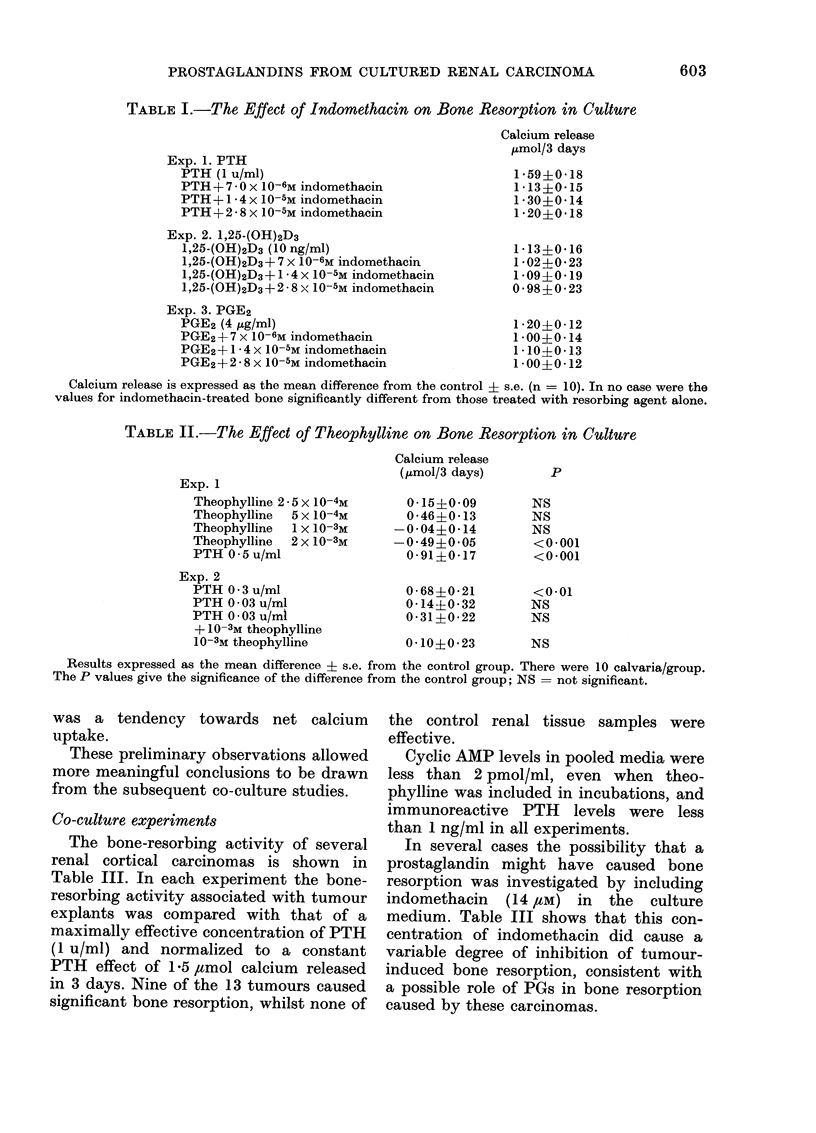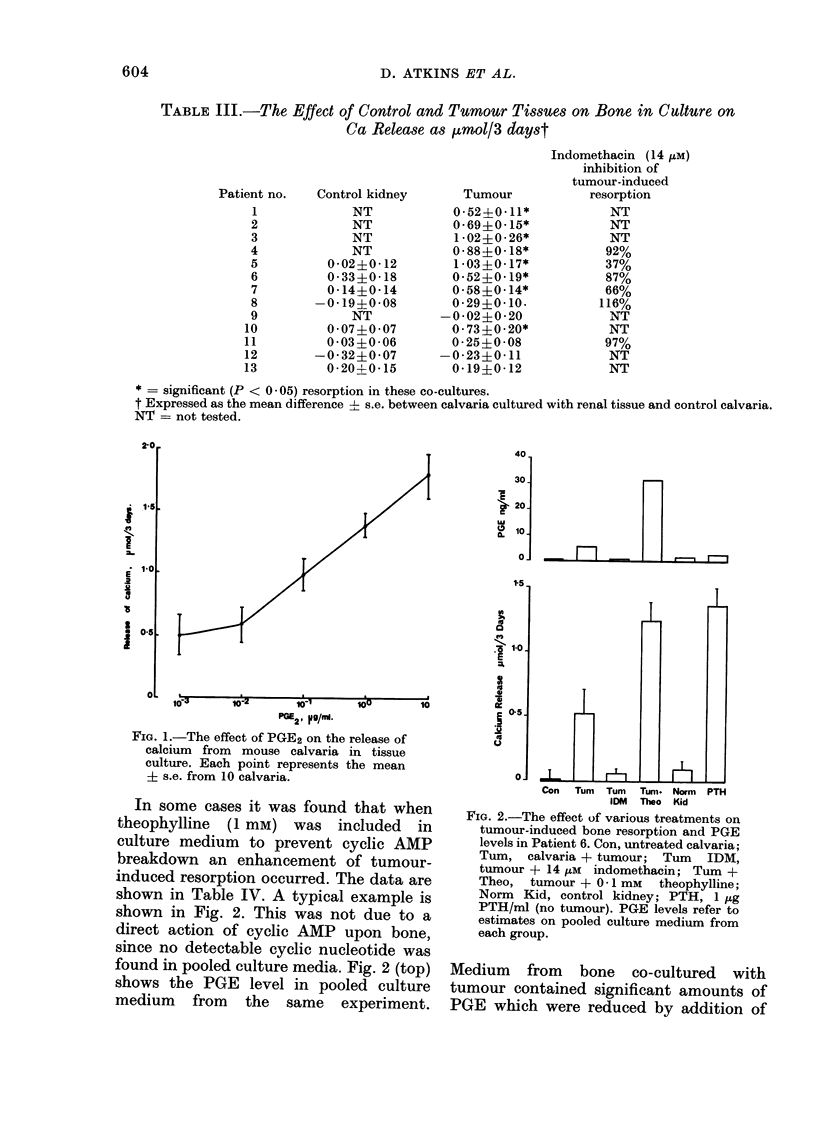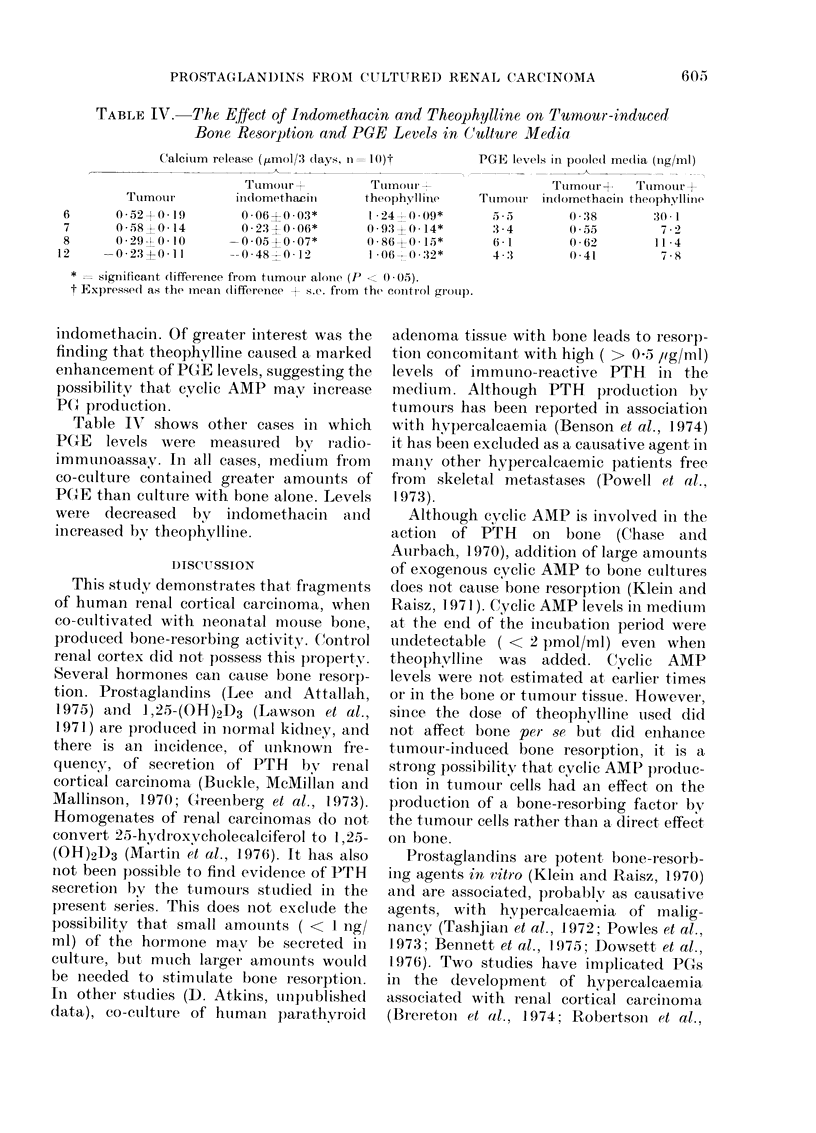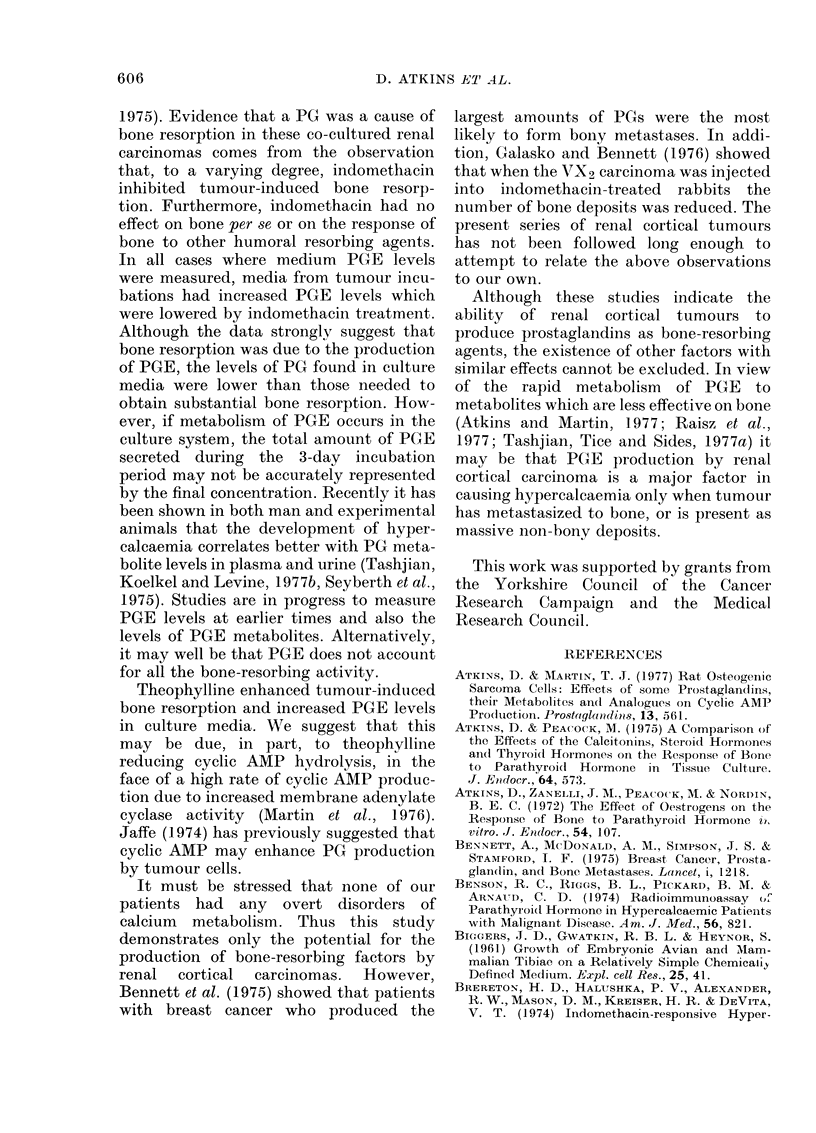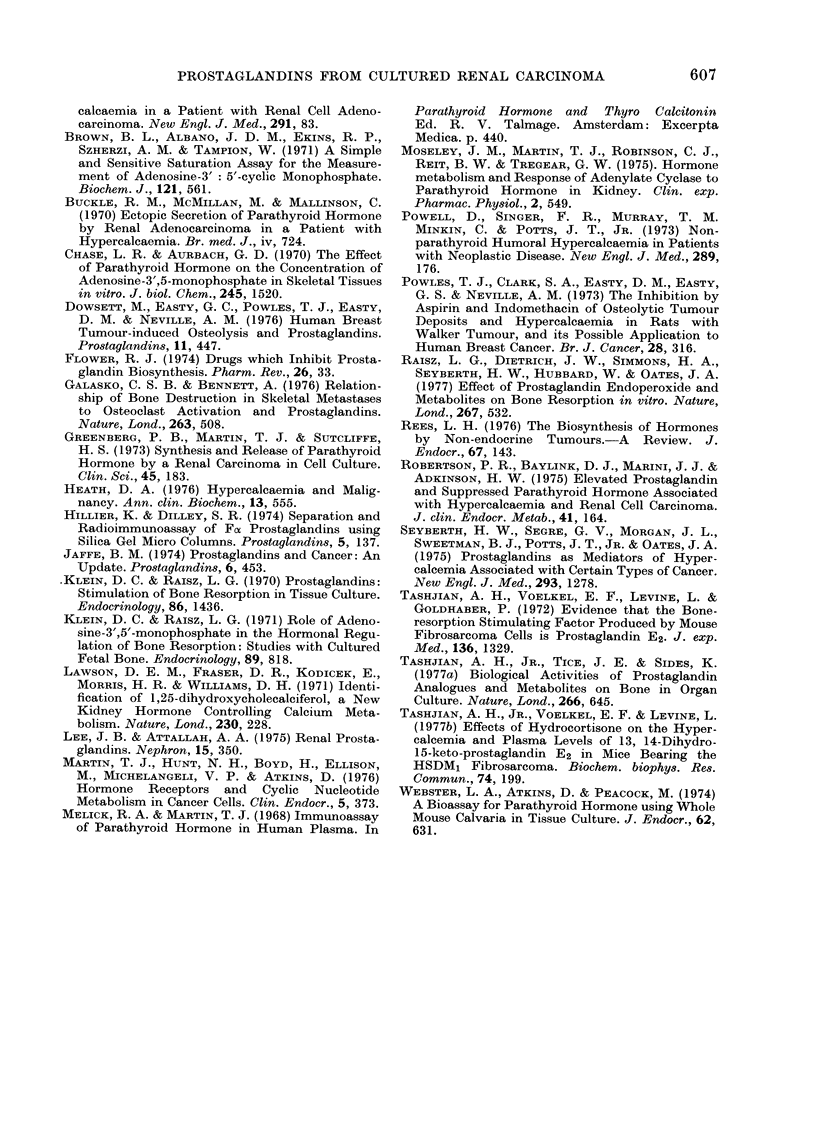# Secretion of prostaglandins as bone-resorbing agents by renal cortical carcinoma in culture.

**DOI:** 10.1038/bjc.1977.237

**Published:** 1977-11

**Authors:** D. Atkins, K. J. Ibbotson, K. Hillier, N. H. Hunt, J. C. Hammonds, T. J. Martin

## Abstract

Fragments of human renal carcinoma tissue have been co-cultured with mouse calvaria. In 9/13 cases significant bone resorption occurred whilst in no case did control kidney cause significant resorption. When bone resorption did occur, it could be reduced by inclusion of indomethacin in the culture medium. In some cases when theophylline was included in culture medium to prevent cyclic AMP breakdown, there was enhancement of tumour-induced bone resorption. Control studies without tumour showed that none of the experimental treatments had a direct effect on bone. Radioimmunoassay of prostaglandin E (PGE) levels in pooled culture media showed that tumour fragments produced appreciable amounts of PGE, and that this production was lowered by indomethacin and increased by theophylline. It is concluded that the bone resorption induced by these tumours is due to a prostaglandin, and that prostaglandin production may be controlled by changes in cyclic AMP metabolism.


*Br. J. Cancer* (1977) **36**, 601

### SECRETION OF PROSTAGLANDINS AS BONE-RESORBING AGENTS BY RENAL CORTICAL CARCINOMA IN CULTURE

D. ATKINS, K. J. IBBOTSON, K. HILLIER*, N. H. HUNT, J. C. HAMMONDS† AND T. J. MARTIN

*From the Department of Chemical Pathology, University of Sheffield Medical School, Sheffield, S10 2RX, the *Department of Clinical Pharmacology, Medical and Biological Sciences, University of Southampton, Southampton, SO9 3TU, and the †Department of Urology, The Royal Hospital, Sheffield*

Received 13 March 1977 Accepted 27 June 1977

**Summary.—Fragments of human renal carcinoma tissue have been co-cultured with mouse calvaria. In 9/13 cases significant bone resorption occurred whilst in no case did control kidney cause significant resorption. When bone resorption did occur, it could be reduced by inclusion of indomethacin in the culture medium. In some cases when theophylline was included in culture medium to prevent cyclic AMP breakdown, there was enhancement of tumour-induced bone resorption. Control studies without tumour showed that none of the experimental treatments had a direct effect on bone. Radioimmunoassay of prostaglandin E (PGE) levels in pooled culture media showed that tumour fragments produced appreciable amounts of PGE, and that this production was lowered by indomethacin and increased by theophylline. It is concluded that the bone resorption induced by these tumours is due to a prostaglandin, and that prostaglandin production may be controlled by changes in cyclic AMP metabolism.**

ADVERSE metabolic effects are frequently associated with both primary and metastatic neoplastic disease, often due to the production by the tumour of a humoral agent (Rees, 1976). Amongst these effects is hypercalcaemia due to the production by neoplastic tissue of bone-resorbing agents, especially when the tumour readily metastasizes to bone.

In the case of renal carcinoma there is a known incidence of hypercalcaemia and also a propensity of the tumour to metastasize to bone (Heath, 1976). Previously it has been shown that the hypercalcaemia may be associated with ectopic parathyroid hormone (PTH) production (Greenberg, Martin and Sutcliffe, 1973) or with prostaglandin (PG) production (Brereton *et al.*, 1974; Robertson *et al.*, 1975).

This paper demonstrates the production of bone-resorbing activity by unselected renal carcinomas in tissue culture, and presents evidence that a PG may be a major causative agent.

### MATERIALS AND METHODS

*Materials.*—BGJ culture medium (Biggers, Gwatkin and Heynor, 1961) was prepared as a powder. The final solution was supplemented with antibiotics, an antifungal agent, ascorbic acid (150 μg/ml) and 15% heated horse serum as described previously (Webster, Atkins and Peacock, 1974).

The PTH used was partially purified bovine hormone with a potency of 1000 u/mg (Moseley *et al.*, 1975), 1,25-dihydroxycholecalciferol (1,25-$(OH)_2D_3$) was the gift of Roche Products Ltd, Welwyn Garden City, and prostaglandin $E_2$ ($PGE_2$) the gift of Dr J. E. Pike, Upjohn Co., Kalamazoo, U.S.A. All other chemicals were obtained from standard suppliers.

Calvaria, for use in bone culture, were obtained from 5–7-day-old Swiss Albino

Correspondence to: Professor T. J. Martin. Department of Medicine, University of Melbourne, Repatriation General Hospital, Heidelberg, 3084, Victoria, Australia.

mice bred in our own colony. Control and tumour tissue was obtained unselectively from patients undergoing nephrectomy for renal cortical carcinoma. All the patients were normocalcaemic; histological examination confirmed the diagnosis in all cases. Tissue, as soon as possible after excision, was placed in ice-cold culture medium for transportation to the laboratory. Explants of material were normally placed in culture within 1·5 h of excision.

*Methods.*—The bone culture method has been described in detail elsewhere (Webster *et al.*, 1974). Briefly, calvaria were explanted and equilibrated in bulk in 40 ml culture medium for 12–24 h at 37 °C in an atmosphere of 5% $CO_2$ in air. After the equilibration period, calvaria were transferred to small culture dishes containing 2 ml of medium. Incubations were then continued for 3 days, at the end of which time the calcium level in the medium was measured by automatic titration using a Corning Model 503 Calcium Analyser.

In co-culture experiments calvaria were placed on the stainless steel grid as usual and surrounded by 4 1-$mm^3$ explants of control or tumour renal tissue. Care was taken to ensure that there was no direct contact between the bone and other tissues. Viability of the explanted renal tissue was assessed by histological examination of the tissue before and after culture. Tissue morphology was preserved with little overt evidence of cell death, thus minimizing the possibility that bone resorption might be produced by components of disintegrating cells.

In experiments where 1,25-$(OH)_2D_3$, $PGE_2$, theophylline or indomethacin were added to culture media, they were dissolved in a small amount of ethanol and added to the medium to give a level of not more than 0·4% ethanol. This level of ethanol has been shown previously not to affect the response of bone to PTH (Atkins *et al.*, 1972). Results were expressed as the release of $\mu$mol calcium during the 3-day incubation period. Differences were assessed by means of Student's *t* test; values of $P < 0{\cdot}05$ were assumed to be significant.

PGE levels were assessed by radioimmunoassay after extraction and purification of pooled media on columns of silicic acid (Hillier and Dilley, 1974). Recovery of added $^3$H-$PGE_2$ was $53{\cdot}6 \pm 2{\cdot}2\%$ (n = 29). The antiserum used did not distinguish between $PGE_1$ and $PGE_2$, and levels are therefore expressed as total PGE (ng/ml) corrected for recovery after silicic acid chromatography. PTH levels were measured by radioimmunoassay (Melick and Martin, 1968) using antiserum BW 211/32 (Burroughs Wellcome) and highly purified bovine PTH for labelling and as standard, and cyclic AMP levels were assessed by a specific protein-binding assay (Brown *et al.*, 1971).

### RESULTS

### *Control experiments*

In order to identify the nature of any bone-resorbing factors produced by tumours, it was necessary to ensure that pharmacological manipulation of the system had effects on tumour cells rather than on bone tissue. The effects of PTH in this bone culture system are well known (Webster *et al.*, 1974) and PTH-induced bone resorption can be prevented by a variety of physiological and pharmacological agents (Atkins and Peacock, 1975). Many of these inhibitors also inhibit bone resorption induced by other agents such as 1,25-$(OH)_2D_3$ (D. Atkins, unpublished observations).

Fig. 1. shows that $PGE_2$ is a potent bone-resorbing agent, the minimum effective dose being 10 ng/ml. Table I shows that bone resorption, however induced, was not inhibited by concentrations of indomethacin which are reported to inhibit prostaglandin biosynthesis (Flower, 1974).

Another sudy has shown (Martin *et al.*, 1976) that membrane adenylate cyclase activity is elevated in renal cortical carcinoma. An attempt was therefore made to reduce adenosine-3′,5′-cyclic monophosphate (cyclic AMP) hydrolysis by including a phosphodiesterase (PDE) inhibitor (theophylline) in culture media. Levels of theophylline generally used to inhibit PDE (10 mM) are toxic to bone (J. N. M. Heersche, personal communication). However, lower levels (up to 2 mM) did not stimulate bone resorption, nor did they modify the responsiveness to PTH (Table II). At the higher dose levels there

TABLE I.—*The Effect of Indomethacin on Bone Resorption in Culture*

| | Calcium release µmol/3 days |
|---|---|
| Exp. 1. PTH | |
| PTH (1 u/ml) | 1·59±0·18 |
| PTH+7·0×10⁻⁶M indomethacin | 1·13±0·15 |
| PTH+1·4×10⁻⁵M indomethacin | 1·30±0·14 |
| PTH+2·8×10⁻⁵M indomethacin | 1·20±0·18 |
| Exp. 2. 1,25-$(OH)_2D_3$ | |
| 1,25-$(OH)_2D_3$ (10 ng/ml) | 1·13±0·16 |
| 1,25-$(OH)_2D_3$+7×$10^{-6}$M indomethacin | 1·02±0·23 |
| 1,25-$(OH)_2D_3$+1·4×$10^{-5}$M indomethacin | 1·09±0·19 |
| 1,25-$(OH)_2D_3$+2·8×$10^{-5}$M indomethacin | 0·98±0·23 |
| Exp. 3. $PGE_2$ | |
| $PGE_2$ (4 µg/ml) | 1·20±0·12 |
| $PGE_2$+7×$10^{-6}$M indomethacin | 1·00±0·14 |
| $PGE_2$+1·4×$10^{-5}$M indomethacin | 1·10±0·13 |
| $PGE_2$+2·8×$10^{-5}$M indomethacin | 1·00±0·12 |

Calcium release is expressed as the mean difference from the control ± s.e. (n = 10). In no case were the values for indomethacin-treated bone significantly different from those treated with resorbing agent alone.

TABLE II.—*The Effect of Theophylline on Bone Resorption in Culture*

| | Calcium release (µmol/3 days) | *P* |
|---|---|---|
| Exp. 1 | | |
| Theophylline 2·5×$10^{-4}$M | 0·15±0·09 | NS |
| Theophylline 5×$10^{-4}$M | 0·46±0·13 | NS |
| Theophylline 1×$10^{-3}$M | −0·04±0·14 | NS |
| Theophylline 2×$10^{-3}$M | −0·49±0·05 | <0·001 |
| PTH 0·5 u/ml | 0·91±0·17 | <0·001 |
| Exp. 2 | | |
| PTH 0·3 u/ml | 0·68±0·21 | <0·01 |
| PTH 0·03 u/ml | 0·14±0·32 | NS |
| PTH 0·03 u/ml +$10^{-3}$M theophylline | 0·31±0·22 | NS |
| $10^{-3}$M theophylline | 0·10±0·23 | NS |

Results expressed as the mean difference ± s.e. from the control group. There were 10 calvaria/group. The *P* values give the significance of the difference from the control group; NS = not significant.

was a tendency towards net calcium uptake.

These preliminary observations allowed more meaningful conclusions to be drawn from the subsequent co-culture studies.

*Co-culture experiments*

The bone-resorbing activity of several renal cortical carcinomas is shown in Table III. In each experiment the bone-resorbing activity associated with tumour explants was compared with that of a maximally effective concentration of PTH (1 u/ml) and normalized to a constant PTH effect of 1·5 µmol calcium released in 3 days. Nine of the 13 tumours caused significant bone resorption, whilst none of the control renal tissue samples were effective.

Cyclic AMP levels in pooled media were less than 2 pmol/ml, even when theophylline was included in incubations, and immunoreactive PTH levels were less than 1 ng/ml in all experiments.

In several cases the possibility that a prostaglandin might have caused bone resorption was investigated by including indomethacin (14 µM) in the culture medium. Table III shows that this concentration of indomethacin did cause a variable degree of inhibition of tumour-induced bone resorption, consistent with a possible role of PGs in bone resorption caused by these carcinomas.

TABLE III.—*The Effect of Control and Tumour Tissues on Bone in Culture on Ca Release as μmol/3 days*†

| Patient no. | Control kidney | Tumour | Indomethacin (14 μM) inhibition of tumour-induced resorption |
|---|---|---|---|
| 1 | NT | 0·52±0·11* | NT |
| 2 | NT | 0·69±0·15* | NT |
| 3 | NT | 1·02±0·26* | NT |
| 4 | NT | 0·88±0·18* | 92% |
| 5 | 0·02±0·12 | 1·03±0·17* | 37% |
| 6 | 0·33±0·18 | 0·52±0·19* | 87% |
| 7 | 0·14±0·14 | 0·58±0·14* | 66% |
| 8 | −0·19±0·08 | 0·29±0·10· | 116% |
| 9 | NT | −0·02±0·20 | NT |
| 10 | 0·07±0·07 | 0·73±0·20* | NT |
| 11 | 0·03±0·06 | 0·25±0·08 | 97% |
| 12 | −0·32±0·07 | −0·23±0·11 | NT |
| 13 | 0·20±0·15 | 0·19±0·12 | NT |

* = significant ($P < 0·05$) resorption in these co-cultures.
† Expressed as the mean difference ± s.e. between calvaria cultured with renal tissue and control calvaria.
NT = not tested.

FIG. 1.—The effect of $PGE_2$ on the release of calcium from mouse calvaria in tissue culture. Each point represents the mean ± s.e. from 10 calvaria.

FIG. 2.—The effect of various treatments on tumour-induced bone resorption and PGE levels in Patient 6. Con, untreated calvaria; Tum, calvaria + tumour; Tum IDM, tumour + 14 μM indomethacin; Tum + Theo, tumour + 0·1 mM theophylline; Norm Kid, control kidney; PTH, 1 μg PTH/ml (no tumour). PGE levels refer to estimates on pooled culture medium from each group.

In some cases it was found that when theophylline (1 mM) was included in culture medium to prevent cyclic AMP breakdown an enhancement of tumour-induced resorption occurred. The data are shown in Table IV. A typical example is shown in Fig. 2. This was not due to a direct action of cyclic AMP upon bone, since no detectable cyclic nucleotide was found in pooled culture media. Fig. 2 (top) shows the PGE level in pooled culture medium from the same experiment. Medium from bone co-cultured with tumour contained significant amounts of PGE which were reduced by addition of

TABLE IV.—*The Effect of Indomethacin and Theophylline on Tumour-induced Bone Resorption and PGE Levels in Culture Media*

| | Calcium release ($\mu$mol/3 days, n = 10)† | | | PGE levels in pooled media (ng/ml) | | |
|---|---|---|---|---|---|---|
| | Tumour | Tumour + indomethacin | Tumour + theophylline | Tumour | Tumour + indomethacin | Tumour + theophylline |
| 6 | 0·52 ± 0·19 | 0·06 ± 0·03* | 1·24 ± 0·09* | 5·5 | 0·38 | 30·1 |
| 7 | 0·58 ± 0·14 | 0·23 ± 0·06* | 0·93 ± 0·14* | 3·4 | 0·55 | 7·2 |
| 8 | 0·29 ± 0·10 | −0·05 ± 0·07* | 0·86 ± 0·15* | 6·1 | 0·62 | 11·4 |
| 12 | −0·23 ± 0·11 | −0·48 ± 0·12 | 1·06 ± 0·32* | 4·3 | 0·41 | 7·8 |

* = significant difference from tumour alone ($P < 0·05$).

† Expressed as the mean difference ± s.e. from the control group.

indomethacin. Of greater interest was the finding that theophylline caused a marked enhancement of PGE levels, suggesting the possibility that cyclic AMP may increase PG production.

Table IV shows other cases in which PGE levels were measured by radioimmunoassay. In all cases, medium from co-culture contained greater amounts of PGE than culture with bone alone. Levels were decreased by indomethacin and increased by theophylline.

### DISCUSSION

This study demonstrates that fragments of human renal cortical carcinoma, when co-cultivated with neonatal mouse bone, produced bone-resorbing activity. Control renal cortex did not possess this property. Several hormones can cause bone resorption. Prostaglandins (Lee and Attallah, 1975) and 1,25-$(OH)_2D_3$ (Lawson *et al.*, 1971) are produced in normal kidney, and there is an incidence, of unknown frequency, of secretion of PTH by renal cortical carcinoma (Buckle, McMillan and Mallinson, 1970; Greenberg *et al.*, 1973). Homogenates of renal carcinomas do not convert 25-hydroxycholecalciferol to 1,25-$(OH)_2D_3$ (Martin *et al.*, 1976). It has also not been possible to find evidence of PTH secretion by the tumours studied in the present series. This does not exclude the possibility that small amounts ( < 1 ng/ml) of the hormone may be secreted in culture, but much larger amounts would be needed to stimulate bone resorption. In other studies (D. Atkins, unpublished data), co-culture of human parathyroid adenoma tissue with bone leads to resorption concomitant with high ( > 0·5 $\mu$g/ml) levels of immuno-reactive PTH in the medium. Although PTH production by tumours has been reported in association with hypercalcaemia (Benson *et al.*, 1974) it has been excluded as a causative agent in many other hypercalcaemic patients free from skeletal metastases (Powell *et al.*, 1973).

Although cyclic AMP is involved in the action of PTH on bone (Chase and Aurbach, 1970), addition of large amounts of exogenous cyclic AMP to bone cultures does not cause bone resorption (Klein and Raisz, 1971). Cyclic AMP levels in medium at the end of the incubation period were undetectable ( < 2 pmol/ml) even when theophylline was added. Cyclic AMP levels were not estimated at earlier times or in the bone or tumour tissue. However, since the dose of theophylline used did not affect bone *per se* but did enhance tumour-induced bone resorption, it is a strong possibility that cyclic AMP production in tumour cells had an effect on the production of a bone-resorbing factor by the tumour cells rather than a direct effect on bone.

Prostaglandins are potent bone-resorbing agents *in vitro* (Klein and Raisz, 1970) and are associated, probably as causative agents, with hypercalcaemia of malignancy (Tashjian *et al.*, 1972; Powles *et al.*, 1973; Bennett *et al.*, 1975; Dowsett *et al.*, 1976). Two studies have implicated PGs in the development of hypercalcaemia associated with renal cortical carcinoma (Brereton *et al.*, 1974; Robertson *et al.*,

1975). Evidence that a PG was a cause of bone resorption in these co-cultured renal carcinomas comes from the observation that, to a varying degree, indomethacin inhibited tumour-induced bone resorption. Furthermore, indomethacin had no effect on bone *per se* or on the response of bone to other humoral resorbing agents. In all cases where medium PGE levels were measured, media from tumour incubations had increased PGE levels which were lowered by indomethacin treatment. Although the data strongly suggest that bone resorption was due to the production of PGE, the levels of PG found in culture media were lower than those needed to obtain substantial bone resorption. However, if metabolism of PGE occurs in the culture system, the total amount of PGE secreted during the 3-day incubation period may not be accurately represented by the final concentration. Recently it has been shown in both man and experimental animals that the development of hypercalcaemia correlates better with PG metabolite levels in plasma and urine (Tashjian, Koelkel and Levine, 1977*b*, Seyberth *et al.*, 1975). Studies are in progress to measure PGE levels at earlier times and also the levels of PGE metabolites. Alternatively, it may well be that PGE does not account for all the bone-resorbing activity.

Theophylline enhanced tumour-induced bone resorption and increased PGE levels in culture media. We suggest that this may be due, in part, to theophylline reducing cyclic AMP hydrolysis, in the face of a high rate of cyclic AMP production due to increased membrane adenylate cyclase activity (Martin *et al.*, 1976). Jaffe (1974) has previously suggested that cyclic AMP may enhance PG production by tumour cells.

It must be stressed that none of our patients had any overt disorders of calcium metabolism. Thus this study demonstrates only the potential for the production of bone-resorbing factors by renal cortical carcinomas. However, Bennett *et al.* (1975) showed that patients with breast cancer who produced the largest amounts of PGs were the most likely to form bony metastases. In addition, Galasko and Bennett (1976) showed that when the $VX_2$ carcinoma was injected into indomethacin-treated rabbits the number of bone deposits was reduced. The present series of renal cortical tumours has not been followed long enough to attempt to relate the above observations to our own.

Although these studies indicate the ability of renal cortical tumours to produce prostaglandins as bone-resorbing agents, the existence of other factors with similar effects cannot be excluded. In view of the rapid metabolism of PGE to metabolites which are less effective on bone (Atkins and Martin, 1977; Raisz *et al.*, 1977; Tashjian, Tice and Sides, 1977*a*) it may be that PGE production by renal cortical carcinoma is a major factor in causing hypercalcaemia only when tumour has metastasized to bone, or is present as massive non-bony deposits.

This work was supported by grants from the Yorkshire Council of the Cancer Research Campaign and the Medical Research Council.

REFERENCES

ATKINS, D. & MARTIN, T. J. (1977) Rat Osteogenic Sarcoma Cells: Effects of some Prostaglandins, their Metabolites and Analogues on Cyclic AMP Production. *Prostaglandins*, **13**, 561.

ATKINS, D. & PEACOCK, M. (1975) A Comparison of the Effects of the Calcitonins, Steroid Hormones and Thyroid Hormones on the Response of Bone to Parathyroid Hormone in Tissue Culture. *J. Endocr.*, **64**, 573.

ATKINS, D., ZANELLI, J. M., PEACOCK, M. & NORDIN, B. E. C. (1972) The Effect of Oestrogens on the Response of Bone to Parathyroid Hormone *in vitro*. *J. Endocr.*, **54**, 107.

BENNETT, A., MCDONALD, A. M., SIMPSON, J. S. & STAMFORD, I. F. (1975) Breast Cancer, Prostaglandin, and Bone Metastases. *Lancet*, i, 1218.

BENSON, R. C., RIGGS, B. L., PICKARD, B. M. & ARNAUD, C. D. (1974) Radioimmunoassay of Parathyroid Hormone in Hypercalcaemic Patients with Malignant Disease. *Am. J. Med.*, **56**, 821.

BIGGERS, J. D., GWATKIN, R. B. L. & HEYNOR, S. (1961) Growth of Embryonic Avian and Mammalian Tibiae on a Relatively Simple Chemically Defined Medium. *Expl. cell Res.*, **25**, 41.

BRERETON, H. D., HALUSHKA, P. V., ALEXANDER, R. W., MASON, D. M., KREISER, H. R. & DEVITA, V. T. (1974) Indomethacin-responsive Hypercalcaemia in a Patient with Renal Cell Adenocarcinoma. *New Engl. J. Med.*, **291**, 83.

BROWN, B. L., ALBANO, J. D. M., EKINS, R. P., SZHERZI, A. M. & TAMPION, W. (1971) A Simple and Sensitive Saturation Assay for the Measurement of Adenosine-3′ : 5′-cyclic Monophosphate. *Biochem. J.*, **121**, 561.

BUCKLE, R. M., McMILLAN, M. & MALLINSON, C. (1970) Ectopic Secretion of Parathyroid Hormone by Renal Adenocarcinoma in a Patient with Hypercalcaemia. *Br. med. J.*, iv, 724.

CHASE, L. R. & AURBACH, G. D. (1970) The Effect of Parathyroid Hormone on the Concentration of Adenosine-3′,5-monophosphate in Skeletal Tissues *in vitro*. *J. biol. Chem.*, **245**, 1520.

DOWSETT, M., EASTY, G. C., POWLES, T. J., EASTY, D. M. & NEVILLE, A. M. (1976) Human Breast Tumour-induced Osteolysis and Prostaglandins. *Prostaglandins*, **11**, 447.

FLOWER, R. J. (1974) Drugs which Inhibit Prostaglandin Biosynthesis. *Pharm. Rev.*, **26**, 33.

GALASKO, C. S. B. & BENNETT, A. (1976) Relationship of Bone Destruction in Skeletal Metastases to Osteoclast Activation and Prostaglandins. *Nature, Lond.*, **263**, 508.

GREENBERG, P. B., MARTIN, T. J. & SUTCLIFFE, H. S. (1973) Synthesis and Release of Parathyroid Hormone by a Renal Carcinoma in Cell Culture. *Clin. Sci.*, **45**, 183.

HEATH, D. A. (1976) Hypercalcaemia and Malignancy. *Ann. clin. Biochem.*, **13**, 555.

HILLIER, K. & DILLEY, S. R. (1974) Separation and Radioimmunoassay of F$\alpha$ Prostaglandins using Silica Gel Micro Columns. *Prostaglandins*, **5**, 137.

JAFFE, B. M. (1974) Prostaglandins and Cancer: An Update. *Prostaglandins*, **6**, 453.

KLEIN, D. C. & RAISZ, L. G. (1970) Prostaglandins: Stimulation of Bone Resorption in Tissue Culture. *Endocrinology*, **86**, 1436.

KLEIN, D. C. & RAISZ, L. G. (1971) Role of Adenosine-3′,5′-monophosphate in the Hormonal Regulation of Bone Resorption: Studies with Cultured Fetal Bone. *Endocrinology*, **89**, 818.

LAWSON, D. E. M., FRASER, D. R., KODICEK, E., MORRIS, H. R. & WILLIAMS, D. H. (1971) Identification of 1,25-dihydroxycholecalciferol, a New Kidney Hormone Controlling Calcium Metabolism. *Nature, Lond.*, **230**, 228.

LEE, J. B. & ATTALLAH, A. A. (1975) Renal Prostaglandins. *Nephron*, **15**, 350.

MARTIN, T. J., HUNT, N. H., BOYD, H., ELLISON, M., MICHELANGELI, V. P. & ATKINS, D. (1976) Hormone Receptors and Cyclic Nucleotide Metabolism in Cancer Cells. *Clin. Endocr.*, **5**, 373.

MELICK, R. A. & MARTIN, T. J. (1968) Immunoassay of Parathyroid Hormone in Human Plasma. In *Parathyroid Hormone and Thyro Calcitonin* Ed. R. V. Talmage. Amsterdam: Excerpta Medica. p. 440.

MOSELEY, J. M., MARTIN, T. J., ROBINSON, C. J., REIT, B. W. & TREGEAR, G. W. (1975). Hormone metabolism and Response of Adenylate Cyclase to Parathyroid Hormone in Kidney. *Clin. exp. Pharmac. Physiol.*, **2**, 549.

POWELL, D., SINGER, F. R., MURRAY, T. M. MINKIN, C. & POTTS, J. T., JR. (1973) Non-parathyroid Humoral Hypercalcaemia in Patients with Neoplastic Disease. *New Engl. J. Med.*, **289**, 176.

POWLES, T. J., CLARK, S. A., EASTY, D. M., EASTY, G. S. & NEVILLE, A. M. (1973) The Inhibition by Aspirin and Indomethacin of Osteolytic Tumour Deposits and Hypercalcaemia in Rats with Walker Tumour, and its Possible Application to Human Breast Cancer. *Br. J. Cancer*, **28**, 316.

RAISZ, L. G., DIETRICH, J. W., SIMMONS, H. A., SEYBERTH, H. W., HUBBARD, W. & OATES, J. A. (1977) Effect of Prostaglandin Endoperoxide and Metabolites on Bone Resorption *in vitro*. *Nature, Lond.*, **267**, 532.

REES, L. H. (1976) The Biosynthesis of Hormones by Non-endocrine Tumours.—A Review. *J. Endocr.*, **67**, 143.

ROBERTSON, P. R., BAYLINK, D. J., MARINI, J. J. & ADKINSON, H. W. (1975) Elevated Prostaglandin and Suppressed Parathyroid Hormone Associated with Hypercalcaemia and Renal Cell Carcinoma. *J. clin. Endocr. Metab.*, **41**, 164.

SEYBERTH, H. W., SEGRE, G. V., MORGAN, J. L., SWEETMAN, B. J., POTTS, J. T., JR. & OATES, J. A. (1975) Prostaglandins as Mediators of Hypercalcemia Associated with Certain Types of Cancer. *New Engl. J. Med.*, **293**, 1278.

TASHJIAN, A. H., VOELKEL, E. F., LEVINE, L. & GOLDHABER, P. (1972) Evidence that the Bone-resorption Stimulating Factor Produced by Mouse Fibrosarcoma Cells is Prostaglandin $E_2$. *J. exp. Med.*, **136**, 1329.

TASHJIAN, A. H., JR., TICE, J. E. & SIDES, K. (1977*a*) Biological Activities of Prostaglandin Analogues and Metabolites on Bone in Organ Culture. *Nature, Lond.*, **266**, 645.

TASHJIAN, A. H., JR., VOELKEL, E. F. & LEVINE, L. (1977*b*) Effects of Hydrocortisone on the Hypercalcemia and Plasma Levels of 13, 14-Dihydro-15-keto-prostaglandin $E_2$ in Mice Bearing the $HSDM_1$ Fibrosarcoma. *Biochem. biophys. Res. Commun.*, **74**, 199.

WEBSTER, L. A., ATKINS, D. & PEACOCK, M. (1974) A Bioassay for Parathyroid Hormone using Whole Mouse Calvaria in Tissue Culture. *J. Endocr.*, **62**, 631.